# Safety, accuracy, and prediction of prognosis in patients with end-stage chronic kidney disease undergoing dobutamine stress cardiac magnetic resonance imaging

**DOI:** 10.3389/fcvm.2023.1228691

**Published:** 2023-08-30

**Authors:** Lukas D. Weberling, Sebastian Seitz, Janek Salatzki, Andreas Ochs, Ailís C. Haney, Deborah Siry, Jannick Heins, Henning Steen, Norbert Frey, Florian André

**Affiliations:** ^1^ Department of Cardiology, Angiology and Pneumology, Heidelberg University Hospital, Heidelberg, Germany; ^2^ DZHK (German Centre for Cardiovascular Research), Partner Site Heidelberg/Mannheim, Germany; ^3^ MVZ-DRZ Heidelberg, Heidelberg, Germany; ^4^ Medneo, Hamburg, Germany

**Keywords:** CMR, cardiovascular imaging, CAD, dialysis, dobutamine, chronic kidney disease

## Abstract

**Introduction:**

Advanced chronic kidney disease (CKD) is an independent risk factor for coronary artery disease (CAD). Due to its unique uremia-derived pathophysiology of atherosclerosis and the limitations of using potentially harmful contrast agents, the best non-invasive approach to assess CAD in these patients remains unclear. We sought to investigate the accuracy, safety, and prognosis of patients with severe CKD undergoing dobutamine stress cardiac magnetic resonance imaging (CMR).

**Materials and methods:**

In this retrospective, single-center study, patients on dialysis or with a glomerular filtration rate of <15 ml/min/1.73 m^2^ who underwent dobutamine stress CMR were included. A rest and stress wall motion analysis was performed using dobutamine/atropine as stressor. The target heart rate was 85% of the maximum heart rate. Periprocedural adverse events and 1-year follow-up data were obtained.

**Results:**

A total of 176 patients (127 men, 49 women) with a mean age of 60.9 ± 14.7 years were included, of which 156 patients were on permanent dialysis. Short-term symptoms such as angina or shortness of breath during stress CMR were frequent (22.1%), but major complications were rare (one patient with myocardial infarction, 0.6%). The 1-year event rate was high (16.4%) with a significant independent correlation to reduced ejection fraction at rest (*p* = 0.037) and failure to achieve the target heart rate (*p* = 0.029). The overall accuracy for predicting significant CAD was good (sensitivity of 71.4%, specificity of 98.4%) and excellent if the target heart rate was achieved (83.3%, 97.9%). A negative stress CMR was highly predictive for the absence of major adverse cardiac event or any coronary revascularization during the 1-year follow-up (negative predictive value of 95.0%).

**Discussion:**

Dobutamine stress CMR is a safe and accurate diagnostic imaging technique in patients at advanced stages of chronic kidney disease. A reduced ejection fraction and the inability to reach the target heart rate are independent predictors of a poor outcome.

## 1. Introduction

Chronic kidney disease (CKD) is an independent risk factor for coronary artery disease (CAD) (1, 2). Patients with CKD are at a three times higher risk for CAD than age- and sex-matched patients with normal renal function (3). Apart from established cardiovascular risk factors (e.g., arterial hypertension, smoking), patients with advanced CKD exhibit a set of uremia-related and paracrine-associated risk factors such as extensive calcification, chronic volume overload, sympathetic overactivity, hyperparathyroidism, oxidative stress, and endothelial dysfunction (4). These specific risk factors reduce the benefit of therapies traditionally applied to patients with CAD. For instance, statin use is not associated with a decreased cardiovascular mortality in patients on dialysis (5, 6). Cardiovascular events remain the leading cause of death even after a successful kidney transplantation, making an accurate diagnostic testing strategy and risk stratification indispensable (7). However, non-invasive imaging for patients with CAD is challenging in patients with advanced CKD. Coronary computed tomography angiography requires iodine-based, potentially nephrotoxic contrast agents and extensive calcifications, a typical and early phenomenon observed in patients with CKD, significantly decrease diagnostic accuracy (8–10). Non-invasive stress tests such as stress echocardiography, nuclear perfusion imaging, and stress cardiac magnetic resonance imaging (CMR) are widely used in patients with CAD, and the optimal diagnostic strategy for CAD in patients with CKD is a subject of current research and scientific discussion (4). To date, stress CMRs are predominantly perfusion examinations using gadolinium-based contrast agents (GBCA) and vasodilators such as adenosine, regadenoson, or dipyridamole to assess myocardial perfusion. Perfusion stress CMR is regarded very safe, and its accuracy is non-inferior to that of the current reference standard, i.e., the invasive fractional flow reserve measurements (11–15). However, reports of the rare but serious nephrogenic systemic fibrosis (NSF) after administering GBCA in patients with advanced CKD (mostly patients on dialysis) have changed clinical practice (16, 17). For that reason, in patients with a glomerular filtration rate (GFR) of <30 ml/min/1.73 m², stress CMR exams are often performed without a contrast agent using the inotropic effect of dobutamine. However, the diagnostic accuracy and periprocedural safety of dobutamine stress CMR in patients with advanced CDK and especially patients on dialysis is currently not fully understood, since only a few studies with small case numbers have investigated the topic (17, 18). A differing underlying pathophysiology for CAD and the extensive comorbidities found in patients with typical advanced CDK lead to the assumption that the excellent safety and diagnostic accuracy of dobutamine stress CMR is possibly not transferable to patients with advanced CDK. This study sought to investigate the safety, accuracy, and predictive power of dobutamine stress CMR in patients with advanced CDK in a high-volume university CMR center.

## 2. Materials and methods

### 2.1. Study population and design

The CMR database was searched for all patients with (i) a dobutamine stress CMR and (ii) chronic kidney failure as defined by a GFR of <15 ml/min/1.73 m^2^ or patients on permanent dialysis for at least 3 months according to the current guidelines (19). In all patients, the hospital information system was searched for complementary information on cardiovascular comorbidities, cardiovascular risk factors, and etiology of kidney failure. As per standard, information with regard to dobutamine dosage, stress CMR results, and vital parameters before, during, and after the exam as well as periprocedural complications are documented for all patients during their CMR. Periprocedural occurrences of symptoms were categorized as symptoms that are mild or moderate (angina, shortness of breath, or palpitations), severe symptoms with a need for medication (beta blockers intravenously or glyceryl trinitrate sublingually), and symptoms with need to abort the exam. Major complications were defined as death, life-threatening arrhythmia, myocardial infarction, or other life-threatening event with potential permanent impact on the health of the patients. The patients were followed up for up to 1 year after the exam, and the outcome was categorized into the following: no event, major adverse cardiac event (MACE) including myocardial infarction or cardiovascular death, major non-cardiac event (e.g., stroke, non-cardiac death), and incomplete follow-up (<12 months had passed since the exam or lost to follow-up). To assess the prognostic impact of stress CMR, a true negative stress exam was assumed if no MACE, no coronary revascularization therapy (stent, bypass), and no invasive coronary angiography with an evidence of a >70% stenosis occurred during the 12 months of follow-up. Similarly, if any of the before-mentioned events did occur, a true positive stress exam was assumed.

### 2.2. CMR acquisition protocol

The examinations were performed on a 1.5 or 3 T MRI scanner (Ingenia and IngeniaCX, Philips Healthcare, Best, Netherlands). For the stress test, cine images of three long axis (two-, three-, four-chamber view) and of three short axis (basal, midventricular, apical) views were obtained using a steady-state free precession with 35 phases per cardiac cycle during a breath-hold. The maximum heart rate for each patient was calculated using the formula HR_max _= 220 – patient age. The target heart rate was defined as ≥85% of the maximum heart rate. The dobutamine dosage was chosen according to weight starting at 10 µg/kg/min and incrementally increased by 10 µg/kg/min every 3 min up to a maximum of 40 µg/kg/min until the target heart rate was reached. If the heart rate response was insufficient (<85% of the maximum heart rate), additional medication of up to 2 mg atropine was used intravenously in the absence of contraindications. The vectorcardiogram, oxygen saturation, and blood pressure of all patients were monitored at any moment, and a cardiologist was always present. The patients were able to communicate with the technician or doctor during the exam via intercom and were regularly asked for the occurrence of symptoms. Stress testing was aborted in the incidence of severe, non-tolerable symptoms such as chest pain or dyspnea, a decrease in systolic blood pressure of >40 mmHg, hypertension of >220/120 mmHg, severe arrhythmias, or an evidence of a positive stress result. A positive stress result was defined as new or worsening wall motion abnormality in ≥1 segment according to literature (20).

### 2.3. Ethical approval

This retrospective study was approved by the Ethical Committee of the University of Heidelberg (S-151/2019). Due to the retrospective nature of the study, the ethical committee did not require an additional informed consent.

### 2.4. Statistical analysis

Analyses were carried out using the R language and environment for statistical computing (version 4.2.1) with the user interface R Studio (version 2022.07.0/548) (21). Normal distribution was assessed using the Shapiro–Wilk test. Parametric variables are given as mean ± standard deviation (SD), and non-parametric variables are given as median with interquartile range. For the comparison of normally distributed variables between two groups, the Welch two sample *t*-test was used. Non-parametrically distributed variables were tested for differences using the Wilcoxon rank-sum test. To analyze the survival probability, a log-rank test (single predictors) and a cox proportional hazard model (multiple predictors: target heart rate achieved, stress result, ejection fraction) were employed, and a Kaplan–Meier estimator was calculated. The *a priori* significance level was set to *p* < 0.05.

## 3. Results

### 3.1. Study population

A total of 18,057 patients were screened, and 176 patients met the inclusion criteria. The study cohort consisted of 127 men and 49 women with a mean age of 60.9 ± 14.7 years. Of those patients, 156 were on permanent dialysis. The underlying pathology for end-stage chronic kidney disease was diabetic nephropathy in 39, hypertensive nephropathy in 17, glomerulonephritis in 50, polycystic kidney disease in 20, tubulointerstitial kidney disease in 13, and other or unknown cause in 37 patients. In 138 patients (78.4%), an invasive coronary angiography was available for direct comparison, which was performed at a median of 123 days (21; 472) before or after CMR. This showed a one-vessel coronary artery disease in 23 patients (15 left anterior descending, five left circumflex, and three right coronary artery), a two-vessel disease in 23 patients, a three-vessel disease in 78 patients, and no coronary artery disease in 14 patients. An involvement of the left main coronary artery was present in 52 patients. The detailed patient characteristics are given in Table 1.

**Table 1:** Overview of the characteristics of all included patients.

Total number of patients	176
Men	127 (72.2%)
Age (years)	60.9 ± 14.7
Undergoing kidney transplantation evaluation	95 (54.0%)
Patients on dialysis	156 (88.6%)
Hemodialysis	134 (76.1%)
Peritoneal dialysis	22 (12.5%)
Risk factors for CAD	
Hypertension	151 (85.8%)
Smoking habit^a^	77 (43.8%)
Hyperlipidemia	80 (45.5%)
Diabetes	78 (44.3%)
Family history	19 (10.8%)
BMI (kg/m^2^)	26.4 ± 4.6
Known CAD	101 (57.4%)
Prior myocardial infarction	45 (25.6%)
Prior percutaneous coronary intervention	66 (37.5%)
Prior coronary bypass surgery	4 (2.3%)
Prior atherosclerosis other than CAD^b^	39 (22.2%)

^a^ Current and ex-smokers.

^b^ Peripheral artery disease or carotid artery stenosis.

### 3.2. CMR results

**Figure 1:**
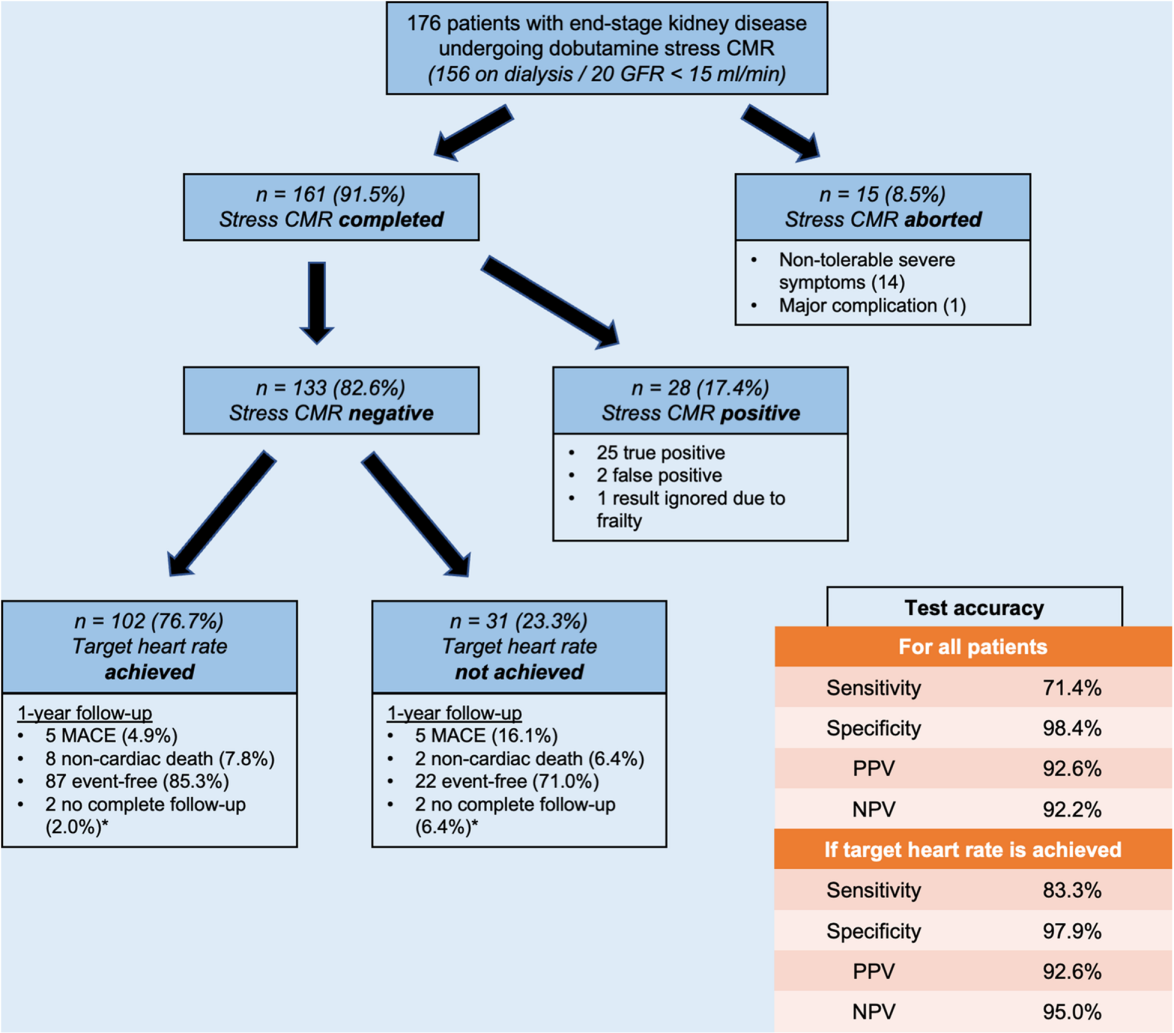
Graphical demonstration of complication rates, achieved target heart rates, event rates, and calculated test accuracy. *These patients were not included in the test accuracy calculation.

**Figure 2:**
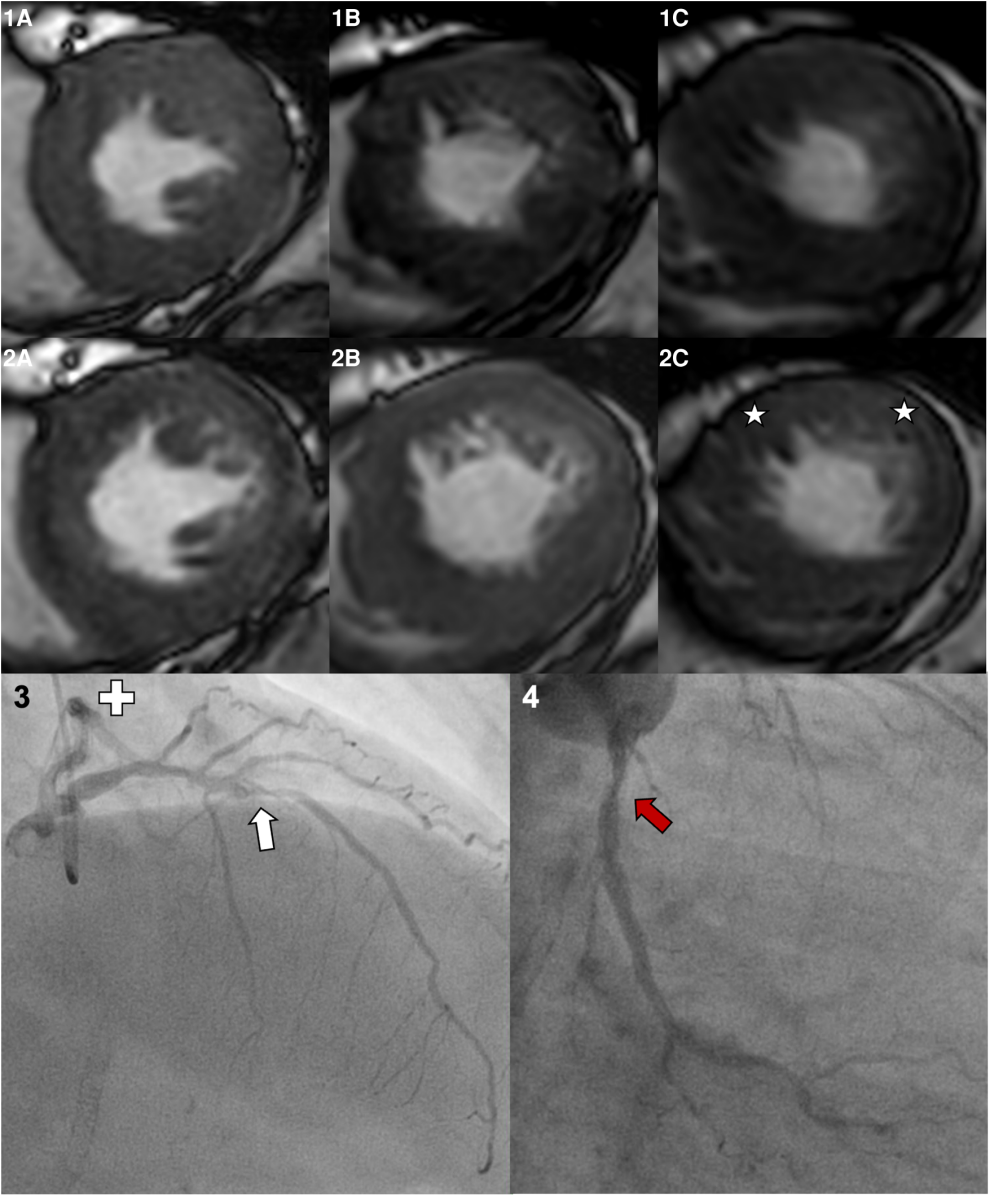
A 55-year-old female patient undergoing dobutamine stress CMR prior to kidney transplantation. Her past medical history included a percutaneous coronary intervention of the right coronary artery with stent implantation but no myocardial infarctions. The LV-EF at rest was good (57%), and the target heart rate of 142/min (86.1% of HF_max_) was reached applying 40 µg/kg/min dobutamine and 0.75 mg atropine. Panel 1 shows the basal (**A**), midventricular (**B**), and apical (**C**) short axis slices at rest. Panel 2 shows images during peak stress, a globally reduced response to peak stress with emphasis on the anterior and lateral wall, and the apical slice (stars) is visible. Consecutive invasive coronary angiography showed a severe stenosis in the left anterior descendent artery (white arrow). The left circumflex artery (Panel 4, separate ostium) shows a moderate coronary stenosis (red arrow). The right coronary artery was inconspicuous beside the previous stent implantation.

An occurrence of new onset of symptoms during stress CMR was frequent (22.1%). Those were tolerable in 5.1% of the patients, needed medication in 9.1% or led to the abortion of the stress exam in 7.9%. The reasons for the abortion of the stress exam were severe angina pectoris in six patients, a hypertensive crisis in four patients, severe shortness of breath in two patients, severe palpitations in two patients, and malaise in two patients. All symptoms diminished minutes after the exam. Major complications were rare with one case of a periprocedural non-ST elevation myocardial infarction (NSTEMI, 0.6%). Stress CMR was successfully completed in 161 patients (91.5%), of which 28 patients (17.4%) had a positive stress result and 133 patients (82.6%) had a negative stress result. A positive stress CMR was highly predictive for the presence of MACE, coronary revascularization, or an evidence of severe coronary artery stenosis (25 true positive patients, positive predictive value of 92.6%). Similarly, a negative stress CMR was highly predictive for the absence of MACE, coronary revascularization, or an evidence of severe coronary artery stenosis for 1 year after the exam (negative predictive value of 92.2%). A coronary revascularization during the 1-year follow-up was necessary in eight of the 133 patients with negative stress CMRs (four NSTEMI, one STEMI, three with high symptom burden and decision for percutaneous coronary intervention despite a negative stress CMR). The predictive value of a negative stress CMR further increased if only the patients with achieved target heart rate were included (a negative predictive value of 95.0%). The testing accuracy of dobutamine stress CMR was excellent if the target heart rate was achieved (sensitivity of 83.3%, specificity of 97.9%), and it was good if the target heart rate was not achieved (sensitivity of 71.4%, specificity of 98.4%). The CMR characteristics, dosages of stress medications, and vital parameters before and during peak stress are given in Table 2. Figure 1 illustrates the different results and stress test accuracy, and Figure 2 shows the exemplary result of a positive stress exam.

**Table 2:** Overview of the cine-derived cardiac measurements of all patients as well as vital signs before and on peak stress.

	Unit	Mean	SD
LVEDD	mm	51.6	7.1
LVESD	mm	34.5	8.9
LVEDV, indexed on BSA	ml/m²	93.7	29.5
LVESV, indexed on BSA	ml/m²	43.3	25.7
LV-EF	%	56.2	12.3
MAPSE	Mm	10.4	3.1
Septum thickness	Mm	13.3	8.1
Lateral wall thickness	Mm	8.0	1.8
LV mass, indexed on BSA	g/m²	71.9	21.9
RVEDD	mm	45.5	7.6
TAPSE	mm	18.6	5.7
Vital signs before stress			
Heart rate	/min	71.3	11.2
BP systolic^a^	mmHg	134.1	24.3
BP diastolic^a^	mmHg	68.3	15.6
Dobutamine dosage	µg/kg	39.0	3.8
Atropine dosage	mg	0.75	0.76
Vital signs at peak stress			
Heart rate	/min	133.1	17.3
BP systolic^b^	mmHg	144.6	37.9
BP diastolic^b^	mmHg	69.8	19.3

LV, left ventricle; LVEDD, LV end-diastolic diameter; LVESD, LV end-systolic diameter; LVEDV, LV end-diastolic volume; BSA, body surface area; LVESV, LV end-systolic volume; LV-EF, LV ejection fraction; MAPSE, mitral annular plane systolic excursion; RVEDD, right ventricular end-diastolic diameter; TAPSE, tricuspid annular plane systolic excursion; BP, blood pressure.

^a^ Available for 174 out of 176 patients.

^b^ Available for 173 out of 176 patients.

### 3.3. Outcome

A 1-year follow-up was obtainable for 171 out of 176 patients (97.2%). Only one patient was lost to follow-up due to relocation to another country. For the remaining four patients, >9 months but <12 months had passed since the exam and the analysis of this study, which is the reason for their exclusion in the outcome evaluation. However, no events had occurred so far in these patients, who all had negative stress CMRs.

The clinical event rate was high in the study group as 16.4% of patients experienced a major cardiac (9.4%) or non-cardiac (7.0%) event. A non-fatal NSTEMI was the main contributor to the cardiac events (4.7%). The 1-year mortality rate was 10.6% (18 patients) with cardiac events accounting for 4.1% (seven patients) of events. A cumulative incidence of adverse events is shown in Table 3.

**Table 3:** Overview of the adverse events during and 12 months after dobutamine stress CMR.

Periprocedural adverse events	40 (22.7%)
Mild/moderate symptoms	9 (5.1%)
Severe symptoms with need for medication^a^	16 (9.1%)
Severe symptoms with abortion of stress exam^b^	14 (7.9%)
Angina pectoris	6 (3.4%)
Hypertensive crisis	4 (2.3%)
Shortness of breath	2 (1.1%)
Palpitations	2 (1.1%)
Malaise	2 (1.1%)
Major complication	1 (0.6%)
Adverse event in the 12 months after CMR	28 (16.4%)
Cardiac death	7 (4.1%)
Major non-cardiac event^c^	12 (7.0%)
Non-fatal NSTEMI	8 (4.7%)
Non-fatal STEMI	1 (0.6%)

^a^ Sublingual glyceryl trinitrate and/or intravenous beta blockers.

^b^ Two patients reported multiple symptoms.

^c^ Defined as non-cardiac death or stroke.

**Figure 3:**
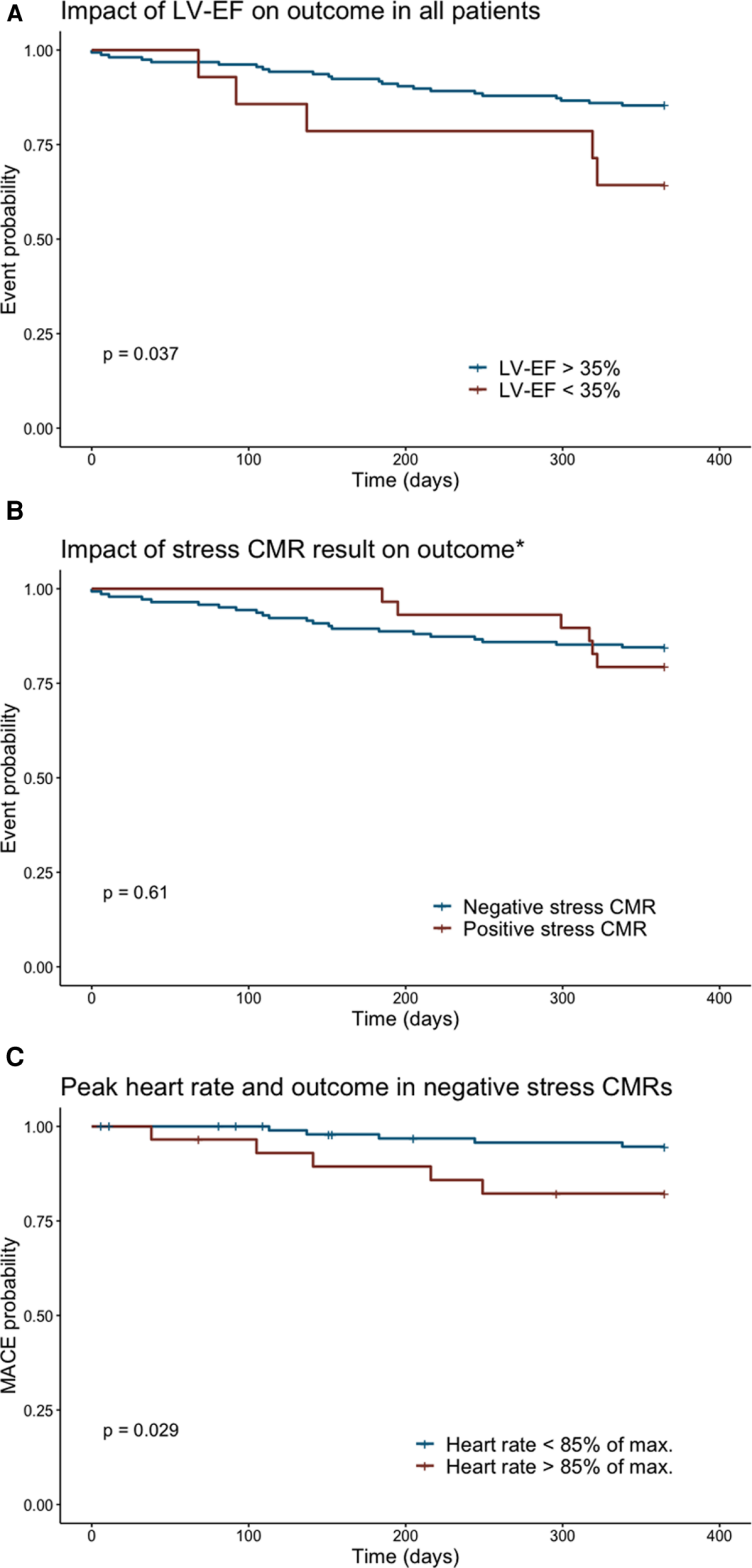
The Kaplan–Meier curves showing the event probability for the cumulative incidence of a major cardiac or non-cardiac event depending on the LV-EF (**A**) or stress result (**B**). (**C**) The occurrence of MACE in negative stress CMRs according to the heart rate response during stress. *Of note, many positive stress CMRs were followed by revascularization therapy. Here, the outcome irrespective of therapy is shown highlighting the positive impact of reperfusion therapy with no significant difference in the outcome to patients with initially negative stress exam.

No differences in the 1-year outcome (MACE and non-cardiac death) were found between patients with aborted vs. completed stress CMR (*p* = 0.51). Similarly, there was no difference in the patient outcome after a positive vs. negative stress result (*p* = 0.61, see Figure 3B). However, the therapeutic regimen differed significantly as the positive stress tests were followed by an invasive revascularization in 13 out of 25 patients. Apart from the stress CMR results, the requests of the patient for further investigations, technical feasibility, symptoms, and expected benefit on the quality of life were among the factors influencing the shared decision making of further treatment. The patients with an invasive revascularization had a significantly better outcome than the patients not undergoing an invasive revascularization despite the positive stress results (*p* = 0.02).

Regarding the multivariate analysis, a left ventricular ejection fraction (LV-EF) of ≤35% at rest was an independent predictor for a poor outcome (non-cardiac and cardiac events) in both the univariate (*p* = 0.037) and multivariate (*p* = 0.047) analysis (see Figure 3A). Equally, the failure to achieve the target heart rate in non-aborted exams was an independent predictor for a poor outcome (see Figure 3C, univariate *p* = 0.029, multivariate *p* = 0.047), and the occurrence of MACE was lowest in the patients with a negative stress result and reached target heart rate (4.9% vs. 16.1%, *p* = 0.03).

## 4. Discussion

Dobutamine stress CMR showed high diagnostic and prognostic accuracy in patients with advanced CKD and to our knowledge this is the largest study in this patient population. Despite its high cardiovascular risk and morbidity, serious adverse events were rare (0.6%) during the CMR scanning, and the diagnostic results were highly predictive for patient outcomes.

The high occurrence rate of cardiac symptoms (22.1%) is linked to a broader definition and thorough documentation by our technicians during the CMR. Those patients experiencing cardiac symptoms that did not lead to the termination of the stress exam are not reported in most comparable studies but comprise most of our reported symptoms (14.1%) (20, 22–24). In a dobutamine stress echocardiography study on 1,118 patients, which reported symptoms without stress exam termination, the observed frequency of angina pectoris was 19.3% and was therefore comparable (25). When juxtaposing the rates of terminated stress exams, the rate of 8.5% in our study on patients with advanced CKD is equivalent to the previous CMR studies reporting rates of 3.0%–11.0% in all-comers (22–24). This is similar for major adverse events such as life-threatening arrhythmias, myocardial infarctions, or death that have been reported in up to 1.0% for all-comers CMR dobutamine stress exams in the literature (20, 22–24, 26). The one major complication (0.6%, myocardial infarction) in the presented study group of patients with advanced CDK is therefore comparable. In the studies also assessing dobutamine stress in patients with advanced CDK, no major adverse events were reported, but the case numbers were too small to account for such rare events (17, 18, 27).

In 23.3% of negative stress CMRs in our study, the target heart rate was not achieved. The data on non-achieved target heart rates vary widely across literature, with a 2004 study by Wahl et al. (22) reporting 9.5% and a 2011 study by Kelle et al. (20) reporting 22.8% of dobutamine stress exams. Despite the varying heart rate response, the diagnostic test accuracy of our study group was good with a sensitivity of 71.4% and specificity of 98.4%. In the multivariate analysis, the inability to reach the target rate was an independent predictor of a poor outcome. Thus, those patients require specific attention by their treating cardiologist. In patients with an adequate heart rate response, the diagnostic accuracy was excellent (sensitivity of 83.3%, specificity of 97.9%) and comparable to all major CMR dobutamine studies assessing all-comers (28). There is only one study assessing the diagnostic accuracy in patients on dialysis showing a sensitivity of 100% and specificity of 89%, but a small case number (47 patients) and a large exclusion rate (24%) limit the comparability to our study (18). To the best of our knowledge, no data exist on the prognostic implications of dobutamine stress CMRs in patients with advanced CDK. However, data does exist for other imaging modalities, and they show that in contrast to other patients with CAD, a large number of patients with advanced CDK with negative test results still experience adverse cardiac outcomes, which is similar to our study (29).

The 1-year cardiac event rate of 4.9% in patients with a negative stress result and achieved target heart rate is noticeably higher than the rate of ∼1.2% reported in large studies for non-CKD patients (20, 23, 30). This reflects the high overall morbidity and mortality of (pre-) dialysis patients. In 2020, the annual mortality rate for all US hemodialysis patients was 186 deaths per 1,000 patient-years with 43% of cardiac causes (31). In contrast, we observed an event rate of 16.4% and death rate of 10.6% for the whole study group. Here, a referral bias with either suspicion of significant CAD or indicated kidney transplantation evaluation (and therefore a dynamic deterioration of health) is most possibly causative for higher event rates, and echocardiography studies on dobutamine stress report equivalent event rates to our study (32). Nevertheless, several predictors influenced event rates. Apart from the abovementioned heart rate response, a reduced LV-EF at rest was also an independent predictor of a poor outcome, an observation that was previously reported for patients on dialysis undergoing dobutamine stress scintigraphy (27).

Several limitations of our study deserve to be discussed. First, the retrospective design of the study has possibly influenced the study results, and therefore prospective studies are needed to confirm the results. Second, the diagnostic accuracy was not compared with other modalities. Here, stress echocardiography and nuclear imaging are the most frequently used alternatives in patients with CKD, but the reported diagnostic accuracy varies widely and hinders direct comparison (4, 33). In line with our study, both have been shown to predict outcome in patients with CKD (29, 34). Third, only dobutamine and not perfusion stress CMR was assessed in our study. Perfusion stress CMR after infusion with adenosine or regadenoson is dependent on GBCA. Despite its better safety profile in comparison with dobutamine, its use has drastically declined in patients with advanced CDK after reports of tissue depositions (e.g., brain) and occurrence of the rare but severe NSF (16, 35, 36). However, both entities are seen almost exclusively after administering linear GBCAs, which are either prohibited (Europe) or whose usage has significantly declined (16, 37, 38). Considering the overall risk of NSF with modern macrocyclic GBCAs (estimated at <0.07%), the usage of perfusion CMR is worth discussing. Apart from that, other medication- and contrast-free CMR protocols have currently been evaluated and might serve as a valuable alternative in patients with CKD (39–42).

In summary, dobutamine stress CMR is a safe and accurate non-invasive imaging modality for CAD risk stratification in patients with advanced CKD and especially in patients on dialysis. A reduced LV-EF and the inability to reach the target heart rate were independent predictors of a poor outcome. The high overall event rate of patients in this study supports the close observation of patients with advanced CDK by an interdisciplinary care team.

## Data Availability

The datasets used and analyzed during the current study are available from the corresponding author on reasonable request.
